# Tattoo sarcoidosis presenting as abdominal allodynia

**DOI:** 10.5694/mja2.51603

**Published:** 2022-06-09

**Authors:** Annabelle Faint, A James M Daveson

**Affiliations:** ^1^ James Cook University Townsville QLD; ^2^ Wesley Medical Research Brisbane QLD; ^3^ Mater Misericordiae Hospital Mackay QLD

**Keywords:** Sarcoidosis, Pain

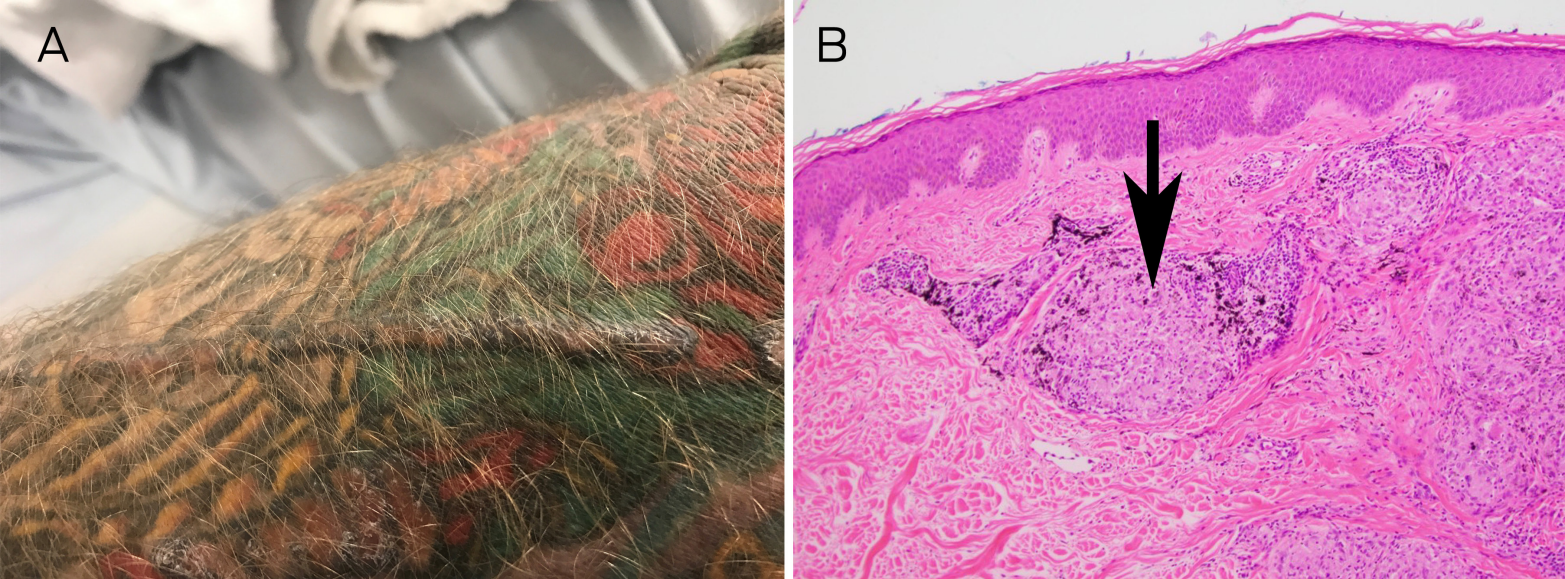

A 40‐year‐old man was referred with left‐sided abdominal pain (described as “like broken‐glass”) radiating across his abdomen, arthralgias and a mild ileitis endoscopically (not histologically). Clinical examination revealed abdominal allodynia and a focally raised, firm and oedematous tattoo (Figure, A). Serum angiotensin converting enzyme (ACE) was 71 IU/L (reference interval [RI], 20–70 IU/L) and ACE mass was 248 μg/L (RI, 37–211 μg/L). A biopsy of the affected tattoo revealed extensive granulomatous dermal inflammation with well defined “naked” tubercles. Polarisation showed a minute amount of exogenous material superficially, although most of the granulomata did not show refractile foreign material, consistent with cutaneous sarcoidosis (Figure, B). Subsequent investigations excluded inflammatory bowel disease. The pain and elevated tattoo resolved with oral corticosteroids (although it initially relapsed when withdrawn) and subsequently methotrexate 20 mg weekly. Sarcoidal reactions to tattoos have been reported^1^ with abdominal pain related to small fibre neuropathies manifest by allodynia and hyperaesthesia.^2^ In one study of patients with cutaneous sarcoidosis treated with methotrexate, lesions completely resolved in 75% of patients.^3^ The prevailing hypothesis is that tattoo pigments provide chronic antigenic stimulation in genetically susceptible patients, leading to systematised granulomatous hypersensitivity.^4^, ^5^

## Open access

Open access publishing facilitated by James Cook University, as part of the Wiley ‐ James Cook University agreement via the Council of Australian University Librarians.

## Competing interests

No relevant disclosures.
